# Impact of Earthquake Hazard on Various Structures—A Case Study in Myanmar

**DOI:** 10.1155/tswj/7539980

**Published:** 2026-08-16

**Authors:** Md. Foisal Haque

**Affiliations:** ^1^ Associate Professor, Department of Civil Engineering, International University of Business Agriculture and Technology, Dhaka, Bangladesh, iubat.edu; ^2^ Geotechnical and Structural Expert, GS Research and Design, Dhaka, Bangladesh

**Keywords:** earthquake on March 28 2025 in Myanmar, incremental dynamic analysis, infrastructure damage assessment, overturning and beam–column joint failure, spectral collapse

## Abstract

Natural disasters cannot be prevented, but it may be possible to reduce their impact on infrastructure by following some guidelines. This study evaluated earthquake‐induced damage (from the March 28, 2025 event in Myanmar) to infrastructure (i.e., reinforced concrete [RC], masonry, and temples) through field investigations and fragility analysis. The overturning and beam–column joint failures with spectral collapse were predicted from the field data, which are presented in terms of several damage states (i.e., slight, moderate, heavy, and very heavy) using incremental dynamic analysis (IDA). Nonlinear time history analysis is used to perform IDA by varying the peak ground acceleration (PGA) in the range of 0–1.5 g. Some issues may influence these failures, such as crack depth, inadequate shear reinforcement at the joint, and inadequate seismic detailing. These recommendations are based on the field inspection. The idealized guidelines are provided to overcome these failures; these include reducing crack depth and placing adequate vertical and horizontal shear reinforcements at joints. This study may be enhanced in the future by including more damage categories and risk analysis.

## 1. Introduction

Surface structures are damaged by natural disasters (i.e., earthquakes or tsunamis) each year in the world. Some of the damage is recoverable, but most of it is not. Many lives may be lost and numerous injuries sustained during an earthquake or tsunami. Recently, a strong earthquake occurred in the Mandalay and Sagaing regions of Myanmar on March 28, 2025. Numerous deaths, injuries, and instances of infrastructure damage were identified from various social media posts. Damage to infrastructure is a vital issue because a hazard to human life is involved in infrastructure performance. For this purpose, the present study performed an IDA based on field data to evaluate the damage probabilities (i.e., slight, moderate, heavy, and very heavy) of structures (i.e., four RC and two masonry) following the recent earthquake.

Recently [1–3], a vulnerability analysis was performed for the RC buildings in the hilly region of India by a rapid visual screening method. The effect of the site‐specific design spectrum of the earthquake‐building parameters was evaluated for the Marmara region in Turkey [4]. The probability of exceedance was evaluated for the structures in the Eastern Anatolian region [5]. The influence of vertical earthquakes on the steel structure was recorded in the previous study [6]. The seismic damage of the masonry structures was evaluated by providing possible solution suggestions [7]. The vulnerability analysis of an RC building with field reconnaissance was performed for the earthquake in Turkey [8]. In addition, the local soil impact on RC structures was evaluated for the seismic excitation [9]. The earthquake damaged the structural performance based on the literature addressed in this section. The damage may occur due to improper construction technique. The spectral collapse, beam–column joint failure, and overturning failure are expressed the damages of structures. Some reasons may be involved behind these damages, such as a column embedded in a retaining wall, using a brick column with the replacement of the RC column, improper alignment of the RC column, and discontinuous load path. These issues were raised in the previous study [3] due to the reason of the improper construction technique, which may lead to damage to structures during hazard scenarios. The landslides are another reason for the damage of structures on the slope. Recently [10], the preliminary assessment of infrastructure in Himachal Pradesh in India was performed to consider the impact of landslides due to heavy rainfall. Another study [11] evaluated the performance of the RC building in the hilly area to consider damage under the sequence of earthquakes. A most recent study [12] studied institutional risk and crisis communication during natural hazards in Yangon City, Myanmar. They mentioned public training for the reduction of risk before a hazard. Another study [13] evaluated the earthquake and tsunami hazards at the Andaman mega‐thrust and Sagaing fault zones. They mentioned a range of historic earthquakes in Myanmar of 7.5–8. Most historic earthquakes occurred at the Mandalay and Bago regions according to their [13] observations. The most recent earthquake on March 28, 2025 occurred in the Mandalay region due to the presence of an active fault line. The simultaneous impact of flood and earthquake was evaluated in the case of railway damage in China [14]. Some portion of the tectonic plate of China may coincide with the Myanmar plate. The damages of structures under the Shen in 1912 and Pyu in 1930 earthquakes were addressed in a previous study [15] for describing the ancient monuments in Chiang Saen in Northern Thailand. The risk analysis of the medical center in Yangon, Myanmar, was performed for the Thabeikkyin earthquake with a magnitude of 6.8 [16]. They assessed the probability of failure for various structures such as hospital buildings, underground tanks, overhead water tanks, and pump houses. A recent study [17] evaluated the damage state of various structures due to the 2014 Mae Lao earthquake in Northern Thailand. The damages to the road, wall of the building, and Gyophyu pipelines under the Taikkyi earthquake in 2017 were described in the previous study [18]. The seismic microzonation map of Mandalay City in Myanmar was addressed in the previous study [13] based on several earthquake records. A recent study [19] mentioned institutions and policy pathways from 1885 to 2015 to mitigate the disaster risk in Myanmar. A most recent study [20] described the hidden seismic hazards in the Golden Triangle region of Laos, Thailand, and Myanmar on the basis of the Sainyabuli earthquake in western Laos in 2019 with a moment magnitude of 6.2. Several studies described the damage to structures due to earthquakes in Myanmar, adjacent China, and Thailand based on the literature addressed in this section.

The main goal of this study is the damage assessment of structures using field observation and IDA. The remedial guidelines are provided based on the observed damage. The damages are classified into three categories: overturning, beam–column joint failure, and spectral collapse. Several deaths and injuries are related to these types of failure of infrastructure. The recorded failures of structures are shown in Table 1, which are found based on past earthquake records in Myanmar. The remedial guidelines with the obtained damage states of infrastructures may help professional engineers to get a preliminary concept of the impact of earthquakes on structures.

**Table 1:** Historical earthquake records in Myanmar [21].

Location	Date	Moment magnitude	Intensity ^ a ^	Focal depth (km)	Deaths	Injuries	Remarks
Sagaing	March 28, 2025	7.7	IX	10	3640+	4570+	Widespread damage
Sagaing	August 6, 1988	7.3	VIII	98.1	3	30	Caused some damage in India and was felt in the Soviet union. At least 30 injured or missing in Bangladesh
Bago	May 5, 1930	7.5	X	35	550–7000	204+	Tsunami
Shan	May 23, 1912	7.8	IX	15–25	/	/	Several cities damaged
Mandalay	March 23, 1839	8.1–8.2	XI	12–15	500+	/	Former capital city Inwa destroyed and abandoned
Chittagong‐Rahkine	April 2, 1762	8.8	XI	/	200+	/	Tsunami

*Note:* “/” means that the information is not available.

^a^Modified Mercalli Intensity Scale.

## 2. Details of Plate Tectonic in Myanmar

**Figure 1:**
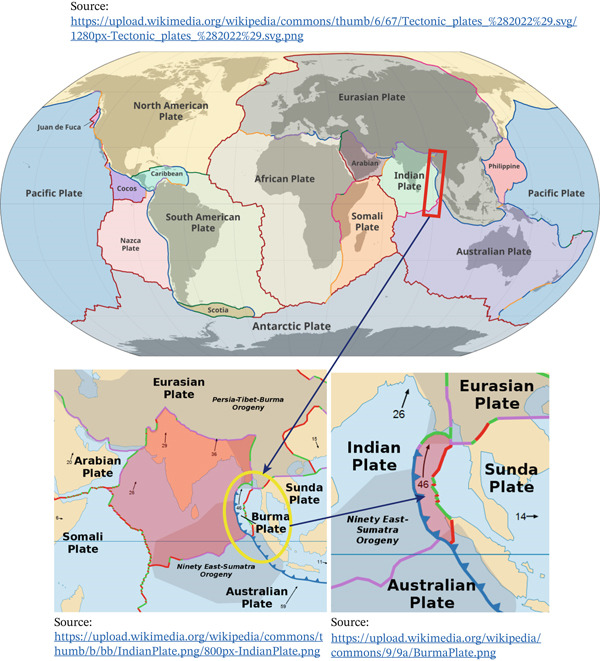
Location of the plate tectonic in Myanmar.

Several tectonic plates are spread across the world, whereas Myanmar stands on the Indian plate, the Burma microplate, and the Shan Thai block. The tectonic setting of Myanmar is adjacent to the Eurasian and Australian plates. The geological location of the Myanmar plate tectonic is depicted in Figure 1. The map presented in Figure 1 is collected from an online source. The movement of the tectonic plates creates fault lines. A huge strain‐energy storage inside the fault line in several locations. The dissipation of the strain energy creates vibrations that are simply called earthquakes. The impact of earthquakes on structures depends on the soil profile, focal depth, epicenter distance, and other things. The right lateral strike‐slip fault is present in central Myanmar from north to south. Most of the historical earthquakes in Myanmar occurred in the Mandalay and Bago regions according to the previous study [13]. This may happen due to the fault line passing through central Myanmar (i.e., Mandalay). The moment magnitude of the historical earthquake stands within a range of 7.5–8. The isoseismal map was prepared based on the movement of the plate tectonic in earthquake histories of 1912 in Shan and 1930 in Pyu at Myanmar, which was used to evaluate structural damages of ancient monuments in Chiang Saen at Northern Thailand [15]. Therefore, the movement of plate tectonics is responsible for the damage to structures. It is not possible to stop or control the movement of plate tectonics because of several reasons (e.g., not visible, lack of instrumental capability, and limited capacity of the human brain). However, it may be possible to minimize the damage to structures due to the movement of plate tectonics by preparing some guidelines. However, the characteristics of the earthquake are gradually changed for several reasons. Therefore, it can be said that natural disasters cannot be prevented by humans using any instrument, but it may be possible to minimize damages and risk due to the impact of disasters (e.g., earthquakes and tsunamis).

## 3. Details of the March 28, 2025 Earthquake in Myanmar

**Figure 2:**
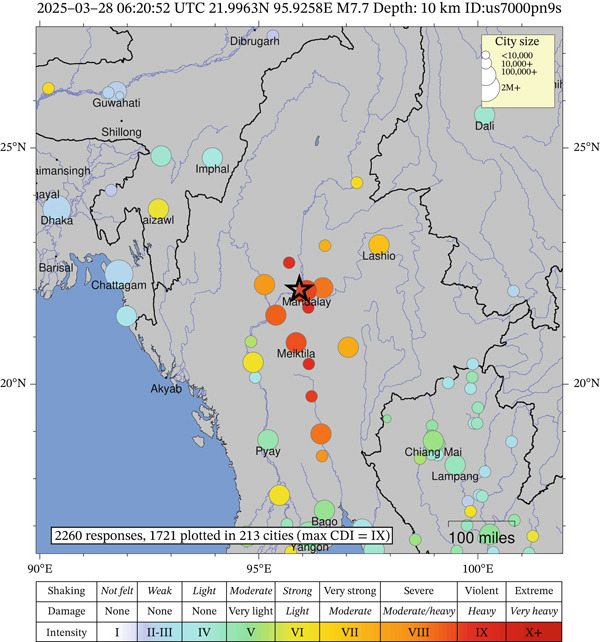
Intensity map of the earthquake on March 28, 2025 in Myanmar (collected from [22]).

The intensity of the earthquake on March 28, 2025 has been recorded at a specific station (i.e., ID: us7000pn9s) with the coordinates of 21.9963 N 95.9258 E [22]. The intensity has been measured using the modified Mercalli scale. The moment magnitude of this earthquake was 7.7 with a focal depth of 10 km. The earthquake occurred at 06:20:52 UTC. The measured intensities in several locations during the earthquake are shown in Figure 2. The epicenter was located at Mandalay, which is located in central Myanmar. The population at Mandalay is more than 2 million based on the recorded data presented in Figure 2. The impact of the earthquake was recorded in 213 cities with 2260 responses. The shaking type was violent, with heavy damage observed. The intensity was measured at the level of IX according to the modified Mercalli scale. Moderate damage was observed at Meiktila with the surrounding area. A light shock was observed close to Chittagong in Bangladesh. The impact of the earthquake was realized in several locations in Myanmar. The size of the right‐lateral fault was 460 × 15 km based on the satellite observations by USGS [22]. Ground failure (i.e., landslides) was observed within significant areas (i.e., more than 100 km^2^). Extensive areas were damaged due to the impact of the liquefaction according to the USGS [22] report. Most of the aboveground building structures were failed by overturning due to the impact of inadequate detailing. Some of the aboveground structures failed due to the spectral collapse of the structural members. This may happen due to the dissipation of strain energy. The earthquake stored a heavy amount of strain energy, which affected the structural members during propagation. Therefore, most of the structural members and structures failed because of the impact of the earthquake on March 28, 2025 in Myanmar. In Mandalay, Myanmar, the soil type is a rock that satisfies the soil type of B based on the Myanmar building code [23]. A minor shock was observed in the Khulna area in Bangladesh based on social media reports. The level of intensity of the March 28, 2025 earthquake in Myanmar was comparatively higher than the previous earthquakes mentioned in the most recent study [20]. Several deaths and injuries have been reported due to this higher intensity and duration. Some data were collected from the waveform report in the USGS [22]. The higher intensity of the earthquake may be responsible for the catastrophic damage to infrastructure in Myanmar.

## 4. Numerical Analysis and Damage Assessment With Remedial Guidelines

**Figure 3:**
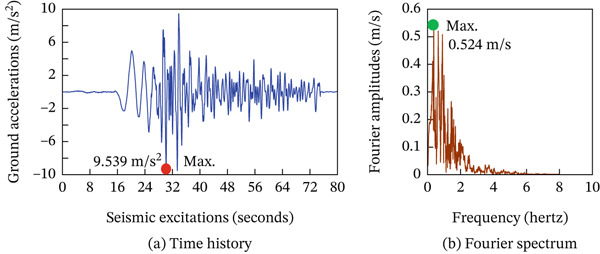
Input ground acceleration and Fourier spectrum. (a) Time history; (b) Fourier spectrum.

**Figure 4:**
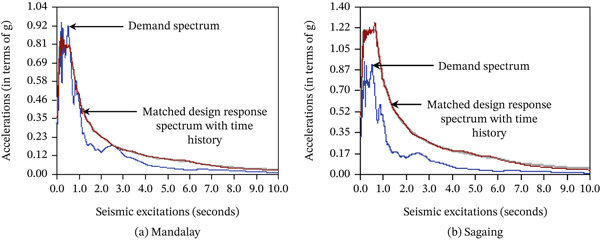
Spectral representation of seismic excitations. (a) Mandalay; (b) Sagaing

This research describes three types of infrastructure failures: overturning, beam–column joint, and spectral collapse. These types of failures may occur due to inadequate design and poor condition of the soil. The design response spectrum is related to the soil classification. The soil is classified into six categories according to the MNBC [23]. The site Class B shows the maximum spectral acceleration. According to the USGS report [22], Mandalay was mostly affected by the impact of the earthquake on March 28, 2025. Therefore, the acceleration record was taken at 900 m away from the central of Mandalay, whereas the latitude and longitude were 19.03^°^ and 109.84^°^. The recorded acceleration and Fourier spectrum are presented in Figure 3a,b, respectively. The IDA is performed to assess several damage states (i.e., slight, moderate, heavy, and very heavy damages) of various structures that are collected from the reported data. These damage states are classified according to the Hazus manual [24] for the RC moment‐resisting frame. In addition, the masonry structure damage states are evaluated using the same manual. The damage state is evaluated in terms of PGA. Details of survey data are presented in Table 2. The IDA is conducted for the nonlinear time history analysis. The recorded ground motion with various PGAs (i.e., 0–1.5 g with 0.1‐g intervals) is used for the IDA. The PGA increment is arbitrarily chosen for the IDA. The IDA is performed using ETABS v. 18.1.1 [25]. The damage states are taken from the manual of EMS [26] and Hazus manual [24]. The field‐observed damages are classified based on the measured displacement during the field visit, which is performed according to the Hazus [24] manual. The damages are subdivided based on changes in PGAs during the IDA according to the Hazus [24] recommendation. These classification techniques are combined to convert the field‐observed damages to the numerical outcomes. The fragility curve indicates the probability of damage state exceedance. The damage state means the exceedance of the plastic hinge rotation of the column at the life safety (LS) level. The strong column‐weak beam theory is implemented in this study, so the column rotation is used to evaluate the overall structural damage. Although the plastic hinge is assigned in the beams and columns in the RC structure. In addition, the plastic hinge is assigned in the masonry wall for the masonry structure. The plastic hinge rotations of the immediate occupancy (IO), LS, and collapse prevention (CP) are addressed in Table 3. The maximum plastic hinge rotation of the column is used to evaluate the damage state for various PGAs. The plastic hinge is assigned near the support for the beam and column. The relative distance is maintained to be 5% (i.e., 0.05 times the column or beam length) from the support. The reported data were collected from the field visit as part of the postdisaster assessment. The damaged buildings due to the impact of the earthquake were discovered during the field visit. The standard format was maintained for the data collection during the field visit. Data are related to the geometric and material properties, in addition to other relevant issues. The material properties of the concrete, steel, and masonry are presented in Table 4. The field data were collected using some instruments, such as a camera, scale, and measurement device. The beam and column are treated as two‐nodded line elements, and the slab is treated as a four‐nodded rectangular element for the numerical analysis. The demand spectrum of Mandalay is comparatively higher than that of Sagaing. The Sagaing demand spectrum is lower than the matched (i.e., design and response) spectrum. The spectral configurations of Mandalay and Sagaing are shown in Figure 4a,b, respectively, which are obtained from the impact of the input motion. The mean, standard deviation, and probability of exceedance are expressed by Equations (1), (2), and (3), respectively, for generating the fragility curves. Site Class B is taken for the development of the response spectrum due to the ground condition of rock in Mandalay and Sagaing. The range of the shear wave velocity for site Class B is 762–1524 m/s. The factors for the short and 1‐s periods of the site Class B are found to be 1.20 and 0.70, respectively, for the Mandalay region. Various damages are evaluated from the crack propagation during the filed visit. The slight, moderate, heavy, and very heavy damages indicate plaster crack, column and beam crack, beam–column joint crack, and larger cracks of structural elements. Various cracks are evaluated from the deflections of the structure and structural members that are found from the numerical analysis. The deflection of the structural member is controlled by the plastic hinge rotation. The three‐dimensional (3D) analysis is performed in ETABS for all structures. The RC building is considered to be a frame structure for the numerical analysis, and the masonry structure is treated as a shell structure during modeling and analysis. The damage states are evaluated to take the stiffness degradation impact of the structural members from the nonlinear time history analysis results by incorporating the forced‐based P‐Delta during the analysis. All gravity and live loads are included in the mass source during the analysis. These loads are taken from the MNBC [23]. The 5% viscous damping is used for the structural members during analysis, and the numerically obtained damages are closely consistent with the observed damages. These variations may influence the strength of the present study.

(1) θ=1ni∑i=1nilnri,

(2) β=1ni−1∑i=1nilnriθ2,

(3) pi=ϕlnri/θβ,

where *n*_*i*_ is the number of plastic hinge rotations of beams and columns; *r*_*i*_ is the number of exceedance of the LS performance level plastic hinge rotations; *θ* is the mean; *β* is the tandard deviation; and *p*_*i*_ is the probability.

**Table 2:** Details of field observations and reported data.

Inspected structures	Framing system	Shape and size	Age (years)	Locations	Damage details
Four‐storied RC building	Moment resisting	Rectangular (18 × 15 m)	30	Mandalay	Flexural cracks were found in beams and columns.
Three‐storied RC building	Moment resisting	Square (15 × 15 m)	35	Sagaing	Several beams and slabs were collapsed during the seismic excitations.
Four‐storied RC building	Moment resisting	L‐shaped (16 × 7.5 × 4/3.5 m)	25	Mandalay	Buckling of reinforcements of the structural members were found by visual observations.
Two‐storied RC building	Moment resisting	Rectangular (7 × 6 m)	35	Mandalay	Beams and columns were completely damaged.
Two‐storied Masonry building	Bearing wall	Square (4.5 × 4.5 m)	50	Sagaing	Slabs were completely collapsed during the seismic excitation, also some parts of wall were damaged.
Single‐storied Temple	Bearing wall	Square (4 × 4 m)	50	Mandalay	Completely destroyed of whole structure.

**Table 3:** Plastic hinge rotations of various components of structures (adopted from [27]).

Components	Plastic rotation angle, radians		
	IO	LS	CP
RC beam	0.0015	0.005	0.01
RC column	0.002	0.005	0.008
Masonry	0.001	0.006	0.008

**Table 4:** Material properties used in the numerical analysis.

Materials	Strength (MPa)	Elastic modulus (MPa)	Poisson′s ratio	Unit weight (kN/m ^ 3 ^ )
Concrete	18 (compressive)	19,940	0.20	24
Steel	415 (yield)	200,000	0.33	77
Masonry	8 (mortar)	5500	0.22	19.5

### 4.1. Overturning Failure With Remedial Measure

The buildings failed due to the overturning of structures, as presented by the probability of exceedance of various damage states. One of the buildings was selected for the evaluation of damage states from the recorded field data. The building was rectangular in shape and failed by overturning. The image of that building is presented in Figure 5a, which was collected from an online source. The main reason for choosing the picture from the online source is the unavailability of the whole picture of the building during the field visit. The plan of this building is shown in Figure 5b. The building is modeled in ETABS for the numerical analysis to evaluate the fragility curves. The 3D configuration with storey height is presented in Figure 5c. In addition, the representation of the plastic hinge assignment in beams and columns is shown in Figure 5d. The sizes of the beam and column, in addition to the slab thickness, are presented in Figure 5b. The material properties of the structural members are addressed in Table 4. The fixed support is considered for the numerical analysis. The compressive strength of the concrete was obtained from the nondestructive test. In addition, the steel strength was found from the visual observation of the grade on the surface of the reinforcement. The compressive strength of concrete may be reduced due to the successive impact of the nonuniform seismic excitation. The presented RC building in Figure 5a has failed due to overturning. The weight of the building was increased due to the placement of an additional rooftop. Furthermore, some of the unnecessary external beams are shown in Figure 5a, which may increase the self‐weight of the structure. The earthquake shock passed through this building. This building is located in Mandalay, which is analyzed for the site Class B specified response spectrum according to the MNBC [23]. Very heavy damage is obtained for this building based on the IDA, and the fragility curve of this building is presented in Figure 5a. Some structural members show slight, moderate, and heavy damage. Various damages are presented by the fragility curves. The range of PGA for the exceedance of very heavy damage is obtained to be 0.8–0.9 g, which is found from the fragility analysis.

Figure 5Geometric configurations with representation of fragility curves of reinforced concrete building. (a) Failure and damage states of reinforced concrete structure; (b) plan of four‐storied RC building; (c) 3D view of the RC building; and (d) sectional elevation with plastic hinges.

**Figure:**
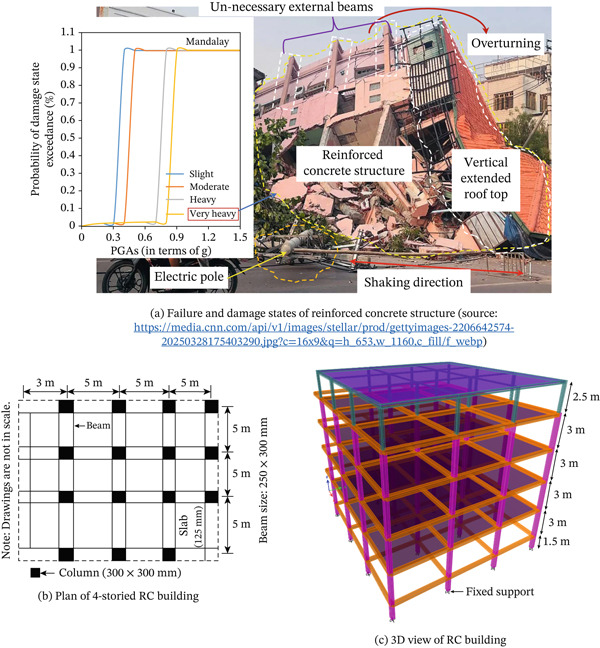
(a)

**Figure:**
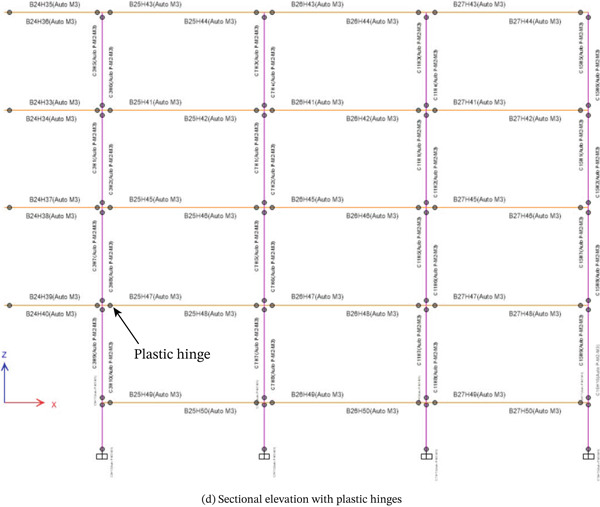
(b)

#### 4.1.1. Remedial Guidelines

The damage due to the overturning may be minimized by considering some techniques for designing the building structures. The depth of cracks in structural members due to the overturning is comparatively higher than in nonstructural components, such as plaster, cable tray, and brick wall. In some cases, the nonstructural components show a higher depth due to the impact of residual strain energy. This happens due to the lower strength of components by using a higher mixing ratio than the standard. It should be minimized by using the code [23] prescribed standard strength and mixing ratio. This type of crack was found in the previous studies [28–31] for the tunnel and piles due to the excessive deflections. It was minimized by increasing the compressive strength of the concrete. Therefore, the damage to RC structural members due to the overturning may be minimized by reducing the crack depth of structural members, joints, and other things. In addition, this failure may be reduced by improving the configuration of reinforcement details of beams and columns, excessive mass effect, irregularity, inadequate lateral resistance, foundation/soil effect, and other things. Maintaining the same level of foundation may reduce the overturning failure. In addition, excessive mass should be removed from the floor level of structures to reduce the overturning failure.

### 4.2. Beam–Column Joint Failure With Remedies

The beam–column joint is a vital part of the building structure. The failure of the joint causes the collapse of the building. The shear failure at the joint happens due to the absence of an additional tie. Sometimes, the tie is provided in one direction, so the shear failure occurs in another direction. Another reason is the spacing of the tie. Some buildings collapsed due to joint failure. Many lives have been lost in addition to several injuries in the earthquake on March 28, 2025. Therefore, one building was chosen during the site visit that failed due to beam–column joint failure, as shown in Figure 6a. The building was 35 years old based on the local people′s opinion that was taken during the site visit. The beam and column sizes with slab thickness are presented in Figure 6b. In addition, the slab thickness and interstorey height are shown in Figure 6c. The used material properties for the nonlinear time history analysis are presented in Table 4. The building was square in size, 15 × 15 m, with a span length of 5 m, as shown in Figure 6b. The failure condition of the RC building with a fragility curve is shown in Figure 6a, which was damaged due to joint failure during seismic shaking. Most joints were heavily fractured due to the inadequate depth and reinforcement in the joint. Several cracks are found in the wall of the building due to the joint failure. In this building, the first and second floors have completely collapsed due to the beam–column joint failure. This happens due to the absence of vertical and horizontal reinforcement layers at the joint. Another reason for the joint failure is the improper bonding of the joint due to a lack of proper tamping during the construction of the building. The impact of the nonuniform seismic excitation shows a higher value at the joint due to the absence of shear reinforcement and proper bonding. The joint takes the compression and tension force at a very close time difference during seismic excitation. Therefore, the hysteresis strain energy is developed inside the joint, and this stored strain energy is dissipated within a short time. This phenomenon may happen due to the joint failure of the building shown in Figure 6a during the earthquake on March 28, 2025 in Myanmar. The failure building is remodeled for the numerical analysis based on the field data, whereas the plastic hinges are located in beams and columns, as shown in Figure 6d.

Figure 6Beam–column joint failure with fragility curves and geometric details of reinforced concrete building. (a) Damage states of beam–column joint failure of RC structure; plan of partial three‐storied RC building; (c) 3D view of the RC building; and (d) sectional elevation with plastic hinge details.

**Figure:**
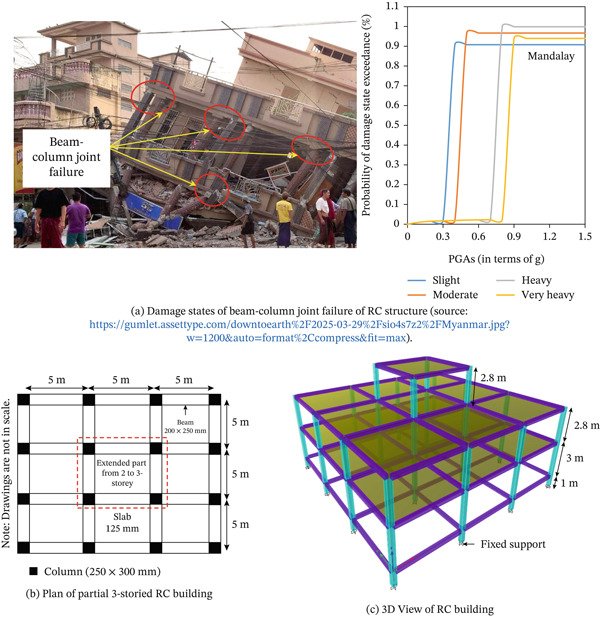
(a)

**Figure:**
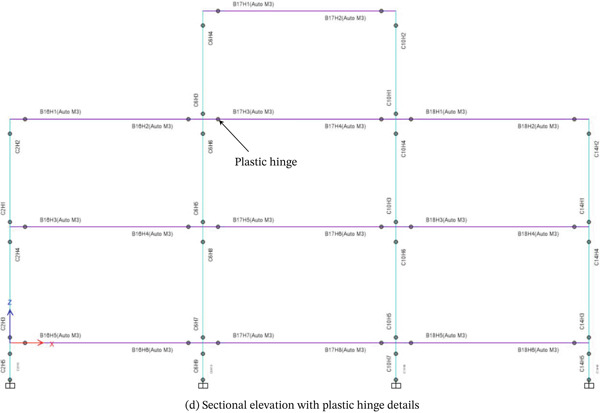
(b)

#### 4.2.1. Remedial Guidelines

The design of the joint should be performed after completing the IDA. The typical joint details should be provided for individual site classes in Myanmar that will help professional engineers with a quick selection of tie reinforcement at the joint, along with its size and spacing. The nonlinear time history analysis may identify the structural performance at the joint during nonuniform seismic excitation. A recent study [32] performed nonlinear static and IDA of the reinforced concrete structure by inserting a plastic hinge at the joint to evaluate the probability of failure at the LS performance level. The evaluation of the hysteresis behavior of the joint is very important for the design. This behavior should be evaluated by using a user‐specified constitutive model that will be created by using a shake table test of the model‐building structure. The shake table is the artificial earthquake testing machine for the model structure in the laboratory. Therefore, it may be required to add detailed explanations of nonlinear static and time history analysis in the MNBC [23] for evaluating the performance of the joint to avoid beam–column joint failure in the future. In addition, improvement of the transverse reinforcements, anchorage, confinement, and seismic detailing of structural members may reduce beam–column joint failure.

### 4.3. Spectral Collapse of Structures With Remedial Guidelines

**Figure 7:**
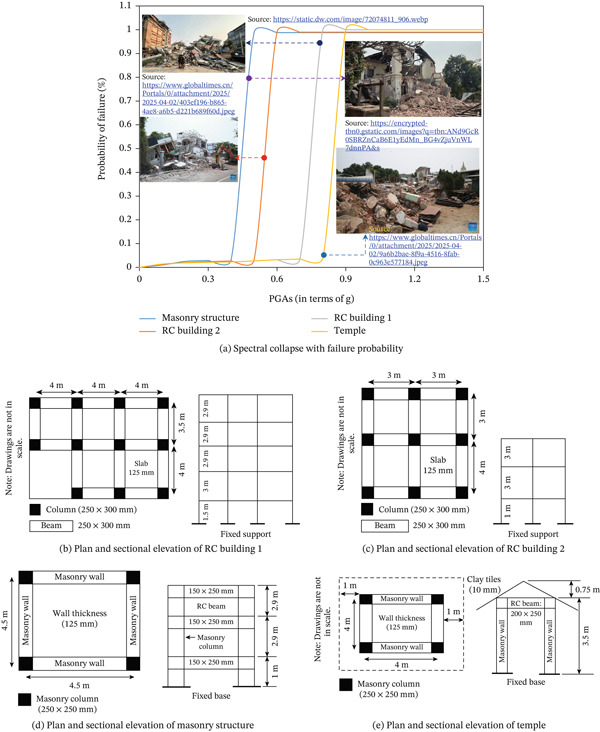
Geometric details and spectral collapse with failure probabilities of various structures. (a) Spectral collapse with failure probability; (b) plan and sectional elevation of RC Building 1; (c) plan and sectional elevation of RC Building 2; (d) plan and sectional elevation of masonry structure; and (e) plan and sectional elevation of temple.

The spectral collapse of structures was found for four types (i.e., masonry structure, reinforced concrete Buildings 1 and 2, and old masonry temple) of the structure during the site visit. Very heavy damage represents this type of failure of structures, although other damages are obtained from the nonlinear time history analysis. The fragility analysis is performed to evaluate the probability of failure for these structures. The fragility curves with damage scenarios are presented in Figure 7a. L‐shaped four‐storied RC structure is presented in Figure 7b, which completely collapsed in Figure 7a. The geometric configuration of a two‐storied RC building is shown in Figure 7c. The beam and column sizes were measured during the field observations. The two‐storied load‐bearing masonry structure is depicted in Figure 7d. The wall thickness was found to be 125 mm from the field inspection. In addition, a single‐bay masonry temple is shown in Figure 7e. These geometric configurations are used in the nonlinear time history analysis. The support condition is considered to be fixed for the numerical analysis. The spectral collapse of the structure may occur for the nonseismic design of structures. The term “spectral collapse” was used in the Hazus manual [24] to evaluate various damage states of structures for the postearthquake assessment. Spectral collapse is ensured from the numerical analysis by varying the degradation of strain energy. This phenomenon may express the reduction of stiffness to cause lateral instability. Another reason of the spectral collapse is the application of improper materials, causing poor quality of construction. Furthermore, engagement of unskilled labor is one of the reasons. In addition, inadequate detailing and reinforcement placement are among the most important reasons for the spectral collapse. Several structures collapsed spectrally during the earthquake on March 28, 2025 in Myanmar at Mandalay. There are several reasons involved in the failure of the RC buildings. Noncompliance with the code for the design of the building is one of them. Normally, masonry buildings are very old in Myanmar, Bangladesh, India, Pakistan, China. The building construction quality of China is comparatively better than that of Myanmar and Bangladesh because of the involvement of expert labor, large‐scale field testing facilities, and other things. The old buildings collapsed due to a lack of code development at that time. The temple is more vulnerable than other structures because of age effects. The old masonry structures are not reinforced; therefore, the seismic force resistance capacity is almost negligible. The failures of various structures for the recent earthquake in Myanmar with fragility curves are shown in Figure 7a. The temple shows a higher probability of failure compared with the other structures. Because the temples were made of masonry, and this structure is very old. Therefore, the seismic force resistance capacity is negligible. In the previous study [15], observed the failure of temples under earthquakes in the Golden Triangle areas in Northern Thailand. Therefore, the abovementioned structures may be saved by following some remedial guidelines.

#### 4.3.1. Remedial Guidelines

All categorized structures must be designed by maintaining the seismic specifications according to the code. The code must incorporate detailed specifications of reinforcement, considerations, and analysis techniques. In addition, the base isolation system may be used in RC and steel buildings to absorb the seismic shock at the base level. The application of reinforced masonry may reduce the level of damage to the structure during seismic excitation. It is mandatory for masonry structures to perform fragility analysis under seismic excitation before implementing it practically. In addition, it may involve using seismic shock‐absorbing bricks in the laboratory. The damper may be attached to the foundation to reduce the seismic shock at the base level. The reinforced masonry temple may be constructed with the replacement of the masonry temple. Another issue is the maintenance and monitoring during and after the construction of any type of structure. The lack of the abovementioned issues may reduce the level of risk of structures under seismic excitation. It is necessary to prepare construction management and safety guidelines, which may help professional engineers during construction. In addition, the reinforced masonry and retrofitted technique of the RC structure may reduce the spectral collapse.

## 5. Conclusions

This research assessed the damages of infrastructures (i.e., four RC and two masonry structures) for the earthquake on March 28, 2025 in Myanmar using field data and fragility analysis. The fragility curves are generated from the IDA results. The IDA is performed by changing PGAs during the nonlinear time history analysis. Three types of failures of infrastructures are discussed in this research, such as overturning, beam–column joint failure, and spectral collapse. These failures are numerically evaluated from the probability of exceedance of the damage states (i.e., slight, moderate, heavy, and very heavy). To do this, the plastic hinge rotation is controlled at the LS level. In addition, remedial measures are explained for these damages. Therefore, the major findings are summarized as follows:

• The overturning failure of RC structures occurred due to the development of crack depth in the structural members during an earthquake. This may happen due to the excessive deflection of the structure. The range of the PGA is found to be 0.8–0.9 g from the fragility curves to express this damage.
• The beam–column joint failure was found due to inadequate shear reinforcement in the joint. Another reason may be inadequate tamping in the joint. Therefore, additional vertical and horizontal shear reinforcements would be placed at the joints of these four RC buildings to reduce the failure.
• The spectral collapse of structures (i.e., two RC and two Masonry) occurred due to inadequate seismic detailing. The RC structure and old temple were completely damaged during the earthquake, which was obtained during the field visit and fragility analysis. The structural members would be designed to maintain proper seismic detailing to overcome this failure.

Some damages with remedial measures are addressed in this research. Therefore, there is scope to extend this research in the future by incorporating more categories of damages, risk analysis, and other things.

## Author Contributions

Md. Foisal Haque: conceptualization, methodology, original writing, resources, reviewing, and editing.

## Funding

No funding was received for this manuscript.

## Ethics Statement

The author has nothing to report.

## Consent

The author has nothing to report.

## Conflicts of Interest

The author declares no conflicts of interest.

## Data Availability

The data that support the findings of this study are available from the corresponding author upon reasonable request.
